# Open Problems in Extracellular RNA Data Analysis: Insights From an ERCC Online Workshop

**DOI:** 10.3389/fgene.2021.778416

**Published:** 2022-01-03

**Authors:** Roger P. Alexander, Robert R Kitchen, Juan Pablo Tosar, Matthew Roth, Pieter Mestdagh, Klaas E. A. Max, Joel Rozowsky, Karolina Elżbieta Kaczor-Urbanowicz, Justin Chang, Leonora Balaj, Bojan Losic, Eric L. Van Nostrand, Emily LaPlante, Bogdan Mateescu, Brian S. White, Rongshan Yu, Aleksander Milosavljevic, Gustavo Stolovitzky, Ryan M. Spengler

**Affiliations:** ^1^ Extracellular RNA Communication Consortium, Phoenix, AZ, United States; ^2^ Cardiovascular Research Center, Massachusetts General Hospital and Harvard Medical School, Charlestown, MA, United States; ^3^ Pasteur Institute of Montevideo and University of the Republic of Uruguay, Montevideo, Uruguay; ^4^ Department of Molecular and Human Genetics, Baylor College of Medicine, Houston, TX, United States; ^5^ Center for Medical Genetics, Department of Biomolecular Medicine, Cancer Research Institute Ghent (CRIG), Ghent University, Ghent, Belgium; ^6^ Laboratory of RNA Molecular Biology, Rockefeller University, New York, NY, United States; ^7^ Department of Molecular Biophysics and Biochemistry, Yale University, New Haven, CT, United States; ^8^ School of Dentistry, University of California, Los Angeles, Los Angeles, CA, United States; ^9^ Department of Neurosurgery, Massachusetts General Hospital and Harvard Medical School, Boston, MA, United States; ^10^ Icahn School of Medicine at Mount Sinai, New York, NY, United States; ^11^ Verna and Marrs McLean Department of Biochemistry and Molecular Biology, Baylor College of Medicine, Houston, TX, United States; ^12^ Brain Research Institute, University of Zurich, Zurich, Switzerland; ^13^ Jackson Laboratory, Bar Harbor, ME, United States; ^14^ Department of Computer Science, Xiamen University, Aginome Scientific, Ltd., Xiamen, China; ^15^ IBM T.J. Watson Research Center, Yorktown Heights, NY, United States; ^16^ School of Medicine and Public Health, University of Wisconsin, Madison, WI, United States

**Keywords:** extracellular RNA, RNA-seq, RNA sequencing, deconvolulion, batch variation, DREAM challenge, tissue of origin, biomarker discovery

## Abstract

We now know RNA can survive the harsh environment of biofluids when encapsulated in vesicles or by associating with lipoproteins or RNA binding proteins. These extracellular RNA (exRNA) play a role in intercellular signaling, serve as biomarkers of disease, and form the basis of new strategies for disease treatment. The Extracellular RNA Communication Consortium (ERCC) hosted a two-day online workshop (April 19–20, 2021) on the unique challenges of exRNA data analysis. The goal was to foster an open dialog about best practices and discuss open problems in the field, focusing initially on small exRNA sequencing data. Video recordings of workshop presentations and discussions are available (https://exRNA.org/exRNAdata2021-videos/). There were three target audiences: experimentalists who generate exRNA sequencing data, computational and data scientists who work with those groups to analyze their data, and experimental and data scientists new to the field. Here we summarize issues explored during the workshop, including progress on an effort to develop an exRNA data analysis challenge to engage the community in solving some of these open problems.

## Introduction

**FIGURE 1:**
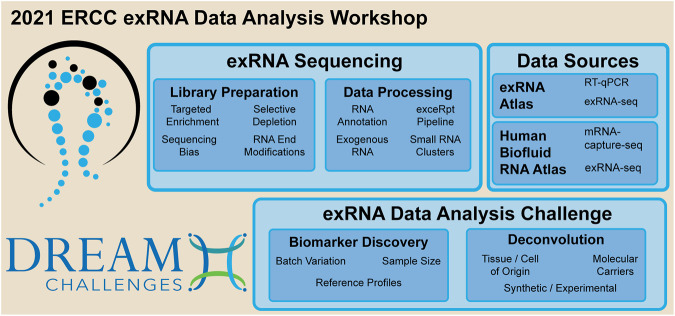
Key topics discussed during the 2021 ERCC exRNA data analysis workshop.

In 2013, the NIH Common Fund launched the Extracellular RNA Communication Consortium to stimulate research into the fundamental biology of exRNA and its clinical applications in disease diagnosis and treatment. One product of the first stage, ERCC1 (2013–2018), is the exRNA Atlas, a database of small RNA-sequencing and RT-qPCR data. To date, the Atlas holds over 7,700 samples from 14 biofluids and 16 disease conditions (Subramanian et al., 2015; Murillo et al., 2019). The current stage, ERCC2, focuses on development of technologies to characterize exRNA carriers and to isolate and characterize the contents of individual extracellular vesicles (EVs). A key strength of the exRNA Atlas is that the small RNA-seq datasets are uniformly processed by the extracellular RNA processing toolkit (exceRpt) (Rozowsky et al., 2019). A hard-learned lesson from ERCC1 was the difficulty of eradicating systematic biases from the data, which makes it difficult to compare exRNA profiles across conditions. The April 2021 online workshop was held to address these and other problems in exRNA data analysis (Figure 1).

## Open Problems in exRNA Data Analysis

Rob Kitchen opened the workshop by outlining open problems in exRNA data analysis. One key challenge is that exRNA data quality varies widely and systematically between different experimental methods. Isolating and purifying exRNA and extracellular vesicles (EVs) from experimental samples is itself difficult, and RNA isolation kits used for these tasks are known to be a major source of variability in the resulting exRNA data. Compensating for this variation is a central challenge, as each kit and RNA sequencing method has different sequence biases that must accounted for when doing larger analyses (Murillo et al., 2019; Srinivasan et al., 2019). Dr. Kitchen stressed that comparison of the relative amounts of exRNAs across samples should be attempted in samples prepared using identical methods wherever possible. Even then, large sample-to-sample variation in exRNA carrier abundance persists, obscuring biological signal in case-control studies (Murillo et al., 2019).

Another central question of interest in exRNA and EV biology is determining tissue and cell type of origin of different clusters of exRNAs in a biofluid. Dr. Kitchen argues that it might be possible in peripheral biofluids like urine and saliva, but more challenging in circulating blood serum and plasma, where the exRNA complement may be too diverse to parse. For vesicular exRNAs, the problem should be made simpler by improvements in experimental techniques to isolate EV sub-fractions, such as selecting for EVs with cell-type-specific surface proteins. As fractionation techniques improve, however, it will be necessary to compensate for variable enrichment efficiency.

Juan Pablo Tosar focused specifically on the quality of non-coding RNA annotations. He outlined how the mechanisms of biogenesis of miRNA and piRNA serve as the basis of existing annotations like miRbase. The problem is that such databases often lack strict curation, resulting in many sequences that are not miRNAs or piRNAs under any reasonable definition. Tosar described two examples from miRbase annotations, showing that miR-1202 is, in fact the small nucleolar RNA, SNORD126, and miR-1246, a microRNA enriched in EVs, is likely a contaminant from fetal bovine serum (FBS) in the cell culture media and likely fragment of the small nuclear RNA RNU2-1 (Sakha et al., 2016; Tosar et al., 2017). This is not a problem of miRbase itself, which is designed as a community-driven repository of putative miRNA sequences, providing minimal quality control at the point of submission (Kozomara et al., 2019). His proposed solution to the problem of mis-annotation is to use a curated miRNA database like MirGeneDB (Fromm et al., 2015; Fromm et al., 2020), resulting in a smaller number of higher quality miRNA calls.

Tosar emphasized that piRNAs have a complex biogenesis that imposes a strong bias to start with U or to have A in the 10th position, and they are expressed from genomic clusters with a high density of piRNA sequences (Czech et al., 2018). PiRNA are mostly expressed in gonads and early embryos where their main role is to dampen the expression of transposable elements. However, existing piRNA databases include a very small number (<1%) of contaminating sequences that do not match these criteria and have 100% overlap with other ncRNA families (Tosar et al., 2018a). Findings of piRNA expression in cancers and biofluids are very often highly enriched in sequences from that set of false positive contaminants (Tosar et al., 2018a). For example, the level of piR-54265 in the serum of colorectal cancer patients has been found to be predictive of tumor relapse after surgery. Its value as a biomarker notwithstanding, piR-54265 is, in fact, a mis-annotated full-length snoRNA, SNORD57 (Tosar et al., 2021). Going back to the cell type of origin problem described above, while piRNA expression might be cancer-specific, snoRNAs are ubiquitously expressed. Consequently, annotation accuracy affects biological interpretation of the data. The final message is that it is important to realize our bioinformatics is only as strong as our RNA annotations.

## exRNA Data Sources

In a session on exRNA data sources, Matt Roth gave an overview of the ERCC’s exRNA Atlas (Subramanian et al., 2015; Murillo et al., 2019), a curated catalog of exRNA sequencing and qPCR data generated from a wide array of biofluids and disease states. Roth outlined features of the exRNA Atlas that facilitate accessing, querying, interpreting, and reusing experimental data and sample metadata. He also described ongoing efforts to expand Atlas content to include data and metadata from additional exRNA technologies being developed as part of ERCC2, and to integrate exRNA Atlas data into the NIH Common Fund Data Ecosystem (https://app.nih-cfde.org/). Justin Chang gave a preview of the exRNA Explorer tool, a data exploration and visualization tool that will soon be integrated into the public exRNA Atlas. Joel Rozowsky later outlined the exceRpt pipeline used to process short exRNA sequencing data in the exRNA Atlas (Rozowsky et al., 2019).

Pieter Mestdagh presented the Human Biofluid RNA Atlas (Hulstaert et al., 2020), which characterizes and compares exRNA transcriptome profiles in a wide variety of biological fluids (*n* = 20) using both small RNA-sequencing and mRNA-capture sequencing. Introducing short and long synthetic spike-in RNAs before and after RNA extraction, enabled comparison of the absolute miRNA and mRNA content between samples, revealing large differences between fluids. Mestdagh also summarized efforts to identify the relative contribution of different tissues to exRNA transcriptomes and how this varied across biofluids. Deconstruction of exRNA profiles into contributing tissues can be achieved through computational deconvolution, a topic explored in-depth later in the workshop. He showed that the accuracy of deconvolution depends on several factors, including 1) proper transformation and normalization of the exRNA-seq read count, 2) choice of deconvolution algorithm and 3) quality and completeness of the reference data. Mestdagh also presented evidence suggesting that circular RNAs (circRNAs) are present in biofluids, potentially at a higher proportional abundance relative to linear transcripts than they are in cells and tissues.

Klaas Max discussed healthy reference profiles of extracellular miRNA in serum and plasma (Max et al., 2018). Based on an initial cohort of 13 individuals and a second larger cohort of over 200 individuals, they found very little variation between males and females, but more noticeable differences between serum and plasma samples. Interestingly, they found that the exRNA profiles of pregnant women were distinct from non-pregnant women, and that the variant miRNAs could predict not only whether a woman were pregnant, but in which stage of pregnancy they were in. Among the most variable miRNAs was cluster-miR-498(46), which was upregulated in pregnant versus non-pregnant women, increasing between 50- and 250-fold from first to third trimester. Max noted that many of the most variable miRNAs in healthy subjects were cell-lineage-specific miRNAs of the liver, neuroendocrine organs, adrenal glands, epithelial cells and muscle. Abundance of several such miRNAs sharing a common origin were moderately correlated. Although they identified additional sets of variably expressed miRNAs, Max noted that few miRNAs are known to be cell-lineage-specific, which complicates methods to deconvolute and identify tissues of origin. Plasma subfractionation by ultracentrifugation to enrich for non-hematopoietic miRNAs did not result in a strong enrichment of organ- or cell-type-specific miRNAs.

## exRNA-Seq Processing

### Exogenous exRNA

Karolina Elżbieta Kaczor-Urbanowicz discussed bioinformatic analysis of salivary RNA sequencing data (Kaczor-Urbanowicz et al., 2018) in the context of a search for exRNA biomarkers of gastric cancer (GC). PI David Wong, co-PI Yong Kim and collaborator Sung Kim in South Korea collected 2000 saliva samples from GC patients and non-GC controls and noticed that saliva has a much higher proportion of microbial RNA than other biofluids. In fact, quality control (QC) criteria for the ERCC’s exceRpt pipeline had to be modified to account for the disproportionately high microbial RNA content. The research team evaluated whether to map RNA-seq reads to microbial RNA before or after mapping to the human genome. They found that the best approach for salivary long RNA-seq data is to map to the microbiome first and remove the aligned bacterial reads before mapping to human. The research team also found that Asian-specific strains of the *Helicobacter pylori* bacteria and Epstein-Barr virus associated with GC for determining disease state when analyzing saliva samples of Asian origin. This highlights the need for ensuring ethnic diversity in human genome and microbiome sequences in the age of personalized medicine. More recently, the researchers have found that performing deconvolution and variance partition analyses to isolate extraneous sources of variation improves their ability to identify exRNA biomarkers.

### Considerations for exRNA Library Preparation

Ryan Spengler emphasized that standard small RNA-seq library preparation methods require RNAs to have 5′ phosphate and 3′ hydroxyl groups, but a substantial fraction of exRNAs lack these end chemistries (Giraldez et al., 2019). By incubating the RNA pool with polynucleotide kinase (PNK) before adapter ligation, Spengler showed that exRNA transcriptome profiles markedly changed, which, for example, significantly increased the number of mRNA and lncRNA fragments found in plasma. These fragments likely originate from specific regions of mRNA transcripts that are protected from RNase degradation, and similar regions are protected across individuals. Careful filtering of reads mapping to repetitive and non-human sequences is essential for identification of *bona fide* mRNA fragments. In a longitudinal study of hematopoietic stem cell transplant recipients, mRNA fragments segregated into several distinct temporal co-expression signatures associated with the transcripts’ likely tissue of origin (namely, liver and bone marrow).

### Biomarker Discovery

Leonora Balaj discussed efforts to identify extracellular mRNA signatures associated with glioma. They examined long RNA-seq reads from EVs isolated from glioma patients compared to healthy individuals matched by age and sex. They also demonstrated a two-hybrid capture method which uses exome capture panels to enrich for exRNA reads from protein-coding mRNAs. Including a ribosomal RNA depletion step, they were able to substantially enrich for mRNA sequences and largely eliminate the non-mRNA reads that dominate non-captured libraries. They also performed long mRNA-seq on exosomal RNAs isolated from patients before and after undergoing dacomitinib treatment for recurrent glioblastoma and found exosomal mRNA signatures that distinguished responders from non-responders to the treatment. Importantly, in follow-up validation cohorts, these signatures showed promise in predicting which individuals would respond to treatment.

### *De Novo* Discovery of Small RNA Clusters

Bojan Losic presented a method for the discovery of small RNA clusters (smRCs, pronounced smirks) expressed in circulating liver cancer EVs (von Felden et al., 2021). The method is *de novo*, not relying on mapping reads to annotated RNAs. In fact, they found that most expressed smRCs emanate from unannotated genomic regions in a cell-type- and biofluid-specific manner and have EV-specific properties which can be exploited for biomarker discovery. Three such smRCs were found to be strong biomarkers for early-stage liver cancer (hepatocellular carcinoma, HCC), significantly outperforming the clinical surveillance standard in an independent Phase 2 clinical study. These findings raise the possibility of a blood-only, operator independent, minimally invasive liquid biopsy test for HCC.

## exRNA and RNA Binding Proteins

Extracellular RNA that circulates in biofluids must be shielded from the harsh environment, particularly from enzymes that digest RNA (RNases). Some exRNAs are resistant to RNase digestion, e.g., Gly/Glu tRNA fragments that can form stable homo- and hetero-dimers (Tosar et al., 2018b). Other exRNAs are protected inside vesicles or by association with RNA-binding proteins (RBPs). Recent work shows that some cell-surface exRNAs are protected by glycosylation (Flynn et al., 2021). Vesicular exRNAs are the best studied class of exRNAs. RBP-associated exRNAs have been difficult to study because of the delicate protein biochemistry required to isolate and characterize RNA binding sites for each of the hundreds of RBPs in the human genome (Gerstberger et al., 2014). Eric Van Nostrand outlined resources from the Encyclopedia of RNA Elements (ENCORE) to aid in this effort, including validated antibodies and shRNA reagents (Sundararaman et al., 2016). ENCORE experiments systematically characterized aspects of RBP regulation including RBP *in vitro* motifs and RNA interactions in K562 and HepG2 cell lines for over 350 RNA binding proteins (Van Nostrand et al., 2020).

Emily LaPlante described initial work scanning the exRNA Atlas for ENCORE RNA binding sites and establishing the infrastructure to allow Atlas users to study their own regions of interest. Bogdan Mateescu outlined current knowledge about exRNA-associated RBPs (exRBPs) (Fabbiano et al., 2020) as well as experimental challenges to discovering new ones. Then he laid out the ERCC2 PRISM (Purification of exRNA by Immuno-capture and Sorting using Microfluidics) project designed to meet those challenges. The exRBP-HIT bioinformatic pipeline identifies candidate exRBPs by overlapping peak calls from exRNA-seq and RBP eCLIP (enhanced CrossLinking and ImmunoPrecipitation) experiments. Permutation analysis of the data yields a ranked list of the most likely candidates. Initial analysis of data from plasma, saliva, and urine samples from the Van Keuren Jensen lab reassuringly identifies exRBPs known to be associated with particular classes of exRNA, for example RNA silencing factors with miRNA and Ro60 with YRNA. More interesting were new candidate exRBPs targeting extracellular mRNA fragments.

The next step in the PRISM group’s strategy is experimental validation of candidate exRBPs in a model system. After using CRISPR to create knockout strains in the 293T cell line for each candidate exRBP gene, exRNA profiles from wild-type and knockout conditioned media are compared. A case study including knockout lines for 10 genes in the RNA silencing pathway led to changes in expression in classes of exRNA beyond miRNA: tRNA, snRNA, snoRNA, and YRNA. Whereas the bioinformatic pipeline identifies individual sites of exRNA interaction with RBPs, the model system provides a global perspective on the impact of each RBP on expression of all exRNAs and perhaps gives insight into exRBP function. The ERCC2 PRISM group plans to identify over 100 candidate exRBPs and create an atlas of exRNA profiles from the model system exRBP knockout strains.

## Deconvolution

Two major open problems in the field are identifying tissue of origin of the exRNAs in a biofluid and associating them with their molecular carrier, whether that be an RNA binding protein, a lipid like HDL or LDL, or a variety of classes of extracellular vesicle. Computational deconvolution, a method for partitioning a heterogeneous dataset into contributions from different independent constituents, can complement experimental approaches to addressing such challenges. There are two broad classes of deconvolution algorithms—reference-based and reference-free. Reference-based algorithms require a known signature matrix of gene expression profiles from all the cell types or molecular carriers to be separated. Reference-free methods estimate simultaneously both the signature matrix and the relative ratios of each cell type or carrier in the mixture.

In a session on deconvolution, Brian White described a Tumor Deconvolution DREAM Challenge (White et al., 2019) to assess existing and inspire novel methods for deconvolving bulk RNA expression data. DREAM (Dialogue on Reverse Engineering Assessment and Methods) Challenges use crowd-sourcing to address fundamental questions in biomedical research. Challenges are posed by domain experts in concert with DREAM organizers, who are responsible for curating data, defining objective evaluation criteria, and engineering a computational framework for method submission and execution. Recently, Challenges have leveraged a “model-to-data” paradigm (Guinney and Saez-Rodriguez 2018) in which models are executed in the cloud, facilitating reproducible deployment of methods and ensuring that those methods are not overfit to data. The DREAM community includes over 30,000 cross-disciplinary participants who have contributed to more than 60 Challenges resulting in over 100 publications (http://DREAMchallenges.org/).

In the Tumor Deconvolution DREAM Challenge, the organizers provided teams RNA expression profiles from *in vitro* and *in silico* admixtures spanning 14 different immune, stromal, and cancer cell types and asked teams to predict the relative ratios of each cell type in the admixtures. To help assess its potential relevance for deconvolving exRNA data, Dr. White described CIBERSORTx, one of the baseline reference methods used in the Challenge. CIBERSORTx is based on the earlier Cell-type Identification By Estimating Relative Subsets Of RNA Transcripts (CIBERSORT) algorithm (Newman et al., 2015). Both CIBERSORT and CIBERSORTx are reference-based. Given an input matrix of reference gene expression signatures from all cell types expected to be present in a mixture, CIBERSORT uses support-vector regression (SVR) to estimate from bulk RNA sequencing data the ratios of each cell type in the mixture. CIBERSORTx (Newman et al., 2019) is a two-stage deconvolution algorithm, including a batch correction step to reduce technical variation across the single-cell RNA-seq datasets used to develop the signature matrix. Results, including those from CIBERSORTx, are summarized on the DREAM Challenge website (https://www.synapse.org/tumorDeconvolutionChallenge).

Rongshan Yu described the DAISM-DNN algorithm (Data Augmentation through *In Silico* Mixing and Deep Neural Network) used to win the Tumor Deconvolution DREAM Challenge. Dr. Yu explained that the choice of a neural network method was guided by the non-linearity of the input data. He showed that expression levels of different genes vary across cell types in a non-linear way. Neural networks perform better than linear regression in that case. The problem with using DNN is that it requires a very large number of training datasets, on the order of ten thousand. The team’s solution was to augment the existing training datasets by shuffling them together *in silico* with expression data from target cells, either bulk RNAseq from purified cell samples or single-cell RNAseq. Finally, although DNN models are generally considered to be difficult to interpret black boxes, it is possible to apply methods such as the SHapley Additive explanation (SHAP) model from game theory (Lundberg and Lee 2017) to output a ranked list of the genes that contribute most to the deconvolution results from DAISM-DNN (Lin et al., 2021).

Finally, Aleks Milosavljevic described the XDec algorithm for analyzing small RNA-seq datasets in the exRNA Atlas. XDec is a two-stage reference-free deconvolution algorithm that separates exRNAs in a biofluid sample into sets associated with different molecular carriers—extracellular vesicles, lipoproteins HDL and LDL, and three categories of RNA binding proteins (Murillo et al., 2019).

## Formulating an exRNA Data Analysis Challenge

A major goal of the workshop was to lay the foundation for creating an exRNA-themed data analysis challenge. The DREAM challenge framework is ideal for presenting problems in data analysis to the wider scientific community. The last session of the workshop was chaired by Gustavo Stolovitzky, co-founder of the DREAM challenges. He gave a talk asking the question: “Can we use a crowdsourcing challenge to benchmark exRNA transcriptomics analyses?” Building on that foundation, Roger Alexander discussed specific classes of challenges that the community might put forth. The ensuing discussion and a post-workshop survey narrowed the field to two classes of challenge: first, a biomarker discovery challenge, and second, a deconvolution challenge to address two problems: identifying cell type of origin and molecular carrier of groups of exRNAs within a biofluid.

### Biomarker Discovery Challenge

Given a set of case-control experiments for a specific disease, what are the most informative sets of exRNA biomarkers for diagnosing and tracking treatment progress for that disease? One of the most difficult aspects of the challenge is acquiring the number of case-control experiments necessary to have sufficient statistical power for biomarker discovery. Improved batch correction algorithms would ease the problem by making it easier to stitch together datasets from multiple related studies. Given large testing and training datasets, teams would compete to develop algorithms that identify exRNA biomarkers that best discriminate cases of disease from controls. The main challenge would reward algorithms that can solve the batch-to-batch variation problem to combine public datasets into a larger testing dataset. A sub-challenge would select for algorithms that perform well even as the original training dataset is repeatedly sub-sampled to be smaller and less powerful. The authors welcome community participation in this effort.

### Deconvolution Challenge

Is it possible to determine the cell type of origin or molecular carrier of different clusters of exRNAs in a biofluid? These questions can begin to be addressed in a data analysis challenge using synthetic datasets. Beyond improved algorithms, a beneficial outcome of such a challenge would be the creation of better gold standards of cell-, tissue-, and molecular carrier-associated exRNA profiles for use with reference-based deconvolution methods.

Moving beyond a synthetic deconvolution challenge requires the generation of an experimental gold standard, which is more challenging for exRNA than for tumor deconvolution. For tumor deconvolution, 14 cell lines were chosen to mimic a tumor microenvironment. For exRNA, the community must come together to agree on a similar proxy environment, perhaps by collecting culture media from cell lines representing several different tissues, with or without a vesicle purification step before RNA isolation and sequencing. Finding that proxy environment was beyond the scope of the workshop but will be necessary to make possible a non-synthetic exRNA deconvolution challenge.

## Discussion

The workshop introduced experimental and data scientists to the field of exRNA data analysis. The standard for small RNA-seq data is uniform processing by the exceRpt pipeline and storage in the exRNA Atlas. Long extracellular RNA is less thoroughly studied, and methods for its analysis are still under development. It is important to be mindful that standard RNA-seq methods are blind to many RNA base modifications, and mis-annotated RNAs can lead to misinterpretation. Biomarker and other studies requiring comparison across datasets would benefit greatly from improved algorithms to compensate for batch-to-batch variation and other systematic errors.

## Conclusion

With the discovery of new classes of exRNA (Flynn et al., 2021) and development of new types of exRNA-based disease therapies (Segel et al., 2021), now is an exciting time in the field of extracellular RNA research. To better our understanding of exRNA biology and improve our ability to use exRNA in the clinic, we wish to issue a call to action to the scientific community. We are seeking a validation dataset that will enable us to launch an exRNA biomarker discovery challenge. Specifically, we seek small exRNA sequencing data for several hundred cases and controls that can be shared prior to publication. The DREAM challenge “model to data” paradigm will ensure that the data remains private in a secure computing environment and is not shared with challenge participants (Ellrott et al., 2019).

## Data Availability

Publicly available datasets were analyzed in this study. Data sources include the exRNA Atlas (https://exRNA-Atlas.org) and the Human Biofluid RNA Atlas (https://r2.amc.nl). Data from the Tumour Deconvolution DREAM Challenge is available at https://www.synapse.org/#!Synapse:syn15589870/wiki/582446.

## References

[B1] Czech B. Munafò M. Ciabrelli F. Eastwood E. L. Fabry M. H. Kneuss E. (2018). piRNA-Guided Genome Defense: From Biogenesis to Silencing. Annu. Rev. Genet. 52, 131–157. 10.1146/annurev-genet-120417-031441 30476449 PMC10784713

[B2] Ellrott K. Buchanan A. Creason A. Mason M. Schaffter T. Hoff B. (2019). Reproducible Biomedical Benchmarking in the Cloud: Lessons from Crowd-Sourced Data Challenges. Genome Biol. 20, 195. 10.1186/s13059-019-1794-0 31506093 PMC6737594

[B3] Fabbiano F. Corsi J. Gurrieri E. Trevisan C. Notarangelo M. D'Agostino V. G. (2020). RNA Packaging into Extracellular Vesicles: An Orchestra of RNA-Binding Proteins? J. Extracell Vesicles 10, e12043. 10.1002/jev2.12043 33391635 PMC7769857

[B4] Flynn R. A. Pedram K. Malaker S. A. Batista P. J. Smith B. A. H. Johnson A. G. (2021). Small RNAs Are Modified with N-Glycans and Displayed on the Surface of Living Cells. Cell 184, 3109–3124. 10.1016/j.cell.2021.04.023 34004145 PMC9097497

[B5] Fromm B. Billipp T. Peck L. E. Johansen M. Tarver J. E. King B. L. (2015). A Uniform System for the Annotation of Vertebrate microRNA Genes and the Evolution of the Human microRNAome. Annu. Rev. Genet. 49, 213–242. 10.1146/annurev-genet-120213-092023 26473382 PMC4743252

[B6] Fromm B. Domanska D. Høye E. Ovchinnikov V. Kang W. Aparicio-Puerta E. (2020). MirGeneDB 2.0: the Metazoan microRNA Complement. Nucleic Acids Res. 48, D132–D141. 10.1093/nar/gkz885 31598695 PMC6943042

[B7] Gerstberger S. Hafner M. Tuschl T. (2014). A Census of Human RNA-Binding Proteins. Nat. Rev. Genet. 15, 829–845. 10.1038/nrg3813 25365966 PMC11148870

[B8] Giraldez M. D. Spengler R. M. Etheridge A. Goicochea A. J. Tuck M. Choi S. W. (2019). Phospho-RNA-seq: a Modified Small RNA-Seq Method that Reveals Circulating mRNA and lncRNA Fragments as Potential Biomarkers in Human Plasma. EMBO J. 38, e101695. 10.15252/embj.2019101695 31053596 PMC6545557

[B9] Guinney J. Saez-Rodriguez J. (2018). Alternative Models for Sharing Confidential Biomedical Data. Nat. Biotechnol. 36, 391–392. 10.1038/nbt.4128 29734317

[B10] Hulstaert E. Morlion A. Avila Cobos F. Verniers K. Nuytens J. Vanden Eynde E. (2020). Charting Extracellular Transcriptomes in the Human Biofluid RNA Atlas. Cel Rep. 33, 108552. 10.1016/j.celrep.2020.108552 33378673

[B11] Kaczor-Urbanowicz K. E. Kim Y. Li F. Galeev T. Kitchen R. R. Gerstein M. (2018). Novel Approaches for Bioinformatic Analysis of Salivary RNA Sequencing Data for Development. Bioinformatics 34, 1–8. 10.1093/bioinformatics/btx504 28961734 PMC5859991

[B12] Kozomara A. Birgaoanu M. Griffiths-Jones S. (2019). miRBase: from microRNA Sequences to Function. Nucleic Acids Res. 47, D155–D162. 10.1093/nar/gky1141 30423142 PMC6323917

[B13] Lin Y. Li H. Xiao X. Zhang L. Wang K. Yang W. (2021). DAISM-DNN^XMBD^: Highly Accurate Cell Type Proportion Estimation with In Silico Data Augmentation and Deep Neural Networks. bioRxiv. 10.1101/2020.03.26.009308v3 PMC9058910 35510186

[B14] Lundberg S. M. Lee S. I. (2017). “A Unified Approach to Interpreting Model Predictions,” in NIPS'17: Proceedings of the 31st International Conference on Neural Information Processing Systems, Long Beach, CA, December 4–9, 2017, 4768–4777.

[B15] Max K. E. A. Bertram K. Akat K. M. Bogardus K. A. Li J. Morozov P. (2018). Human Plasma and Serum Extracellular Small RNA Reference Profiles and Their Clinical Utility. Proc. Natl. Acad. Sci. USA 115, E5334–E5343. 10.1073/pnas.1714397115 29777089 PMC6003356

[B16] Murillo O. D. Thistlethwaite W. Rozowsky J. Subramanian S. L. Lucero R. Shah N. (2019). exRNA Atlas Analysis Reveals Distinct Extracellular RNA Cargo Types and Their Carriers Present across Human Biofluids. Cell 177, 463–477. 10.1016/j.cell.2019.02.018 30951672 PMC6616370

[B17] Newman A. M. Liu C. L. Green M. R. Gentles A. J. Feng W. Xu Y. (2015). Robust Enumeration of Cell Subsets from Tissue Expression Profiles. Nat. Methods 12, 453–457. 10.1038/nmeth.3337 25822800 PMC4739640

[B18] Newman A. M. Steen C. B. Liu C. L. Gentles A. J. Chaudhuri A. A. Scherer F. (2019). Determining Cell Type Abundance and Expression from Bulk Tissues with Digital Cytometry. Nat. Biotechnol. 37, 773–782. 10.1038/s41587-019-0114-2 31061481 PMC6610714

[B19] Rozowsky J. Kitchen R. R. Park J. J. Galeev T. R. Diao J. Warrell J. (2019). exceRpt: A Comprehensive Analytic Platform for Extracellular RNA Profiling. Cel Syst. 8, 352–357. 10.1016/j.cels.2019.03.004 PMC7079576 30956140

[B20] Sakha S. Muramatsu T. Ueda K. Inazawa J. (2016). Exosomal microRNA miR-1246 Induces Cell Motility and Invasion through the Regulation of DENND2D in Oral Squamous Cell Carcinoma. Sci. Rep. 6, 38750. 10.1038/srep38750 27929118 PMC5144099

[B21] Segel M. Lash B. Song J. Ladha A. Liu C. C. Jin X. (2021). Mammalian Retrovirus-like Protein PEG10 Packages its Own mRNA and Can Be Pseudotyped for mRNA Delivery. Science 373, 882–889. 10.1126/science.abg6155 34413232 PMC8431961

[B22] Srinivasan S. Yeri A. Cheah P. S. Chung A. Danielson K. De Hoff P. (2019). Small RNA Sequencing across Diverse Biofluids Identifies Optimal Methods for exRNA Isolation. Cell 177, 446–462. 10.1016/j.cell.2019.03.024 30951671 PMC6557167

[B23] Subramanian S. L. Kitchen R. R. Alexander R. Carter B. S. Cheung K.-H. Laurent L. C. (2015). Integration of Extracellular RNA Profiling Data Using Metadata, Biomedical Ontologies and Linked Data Technologies. J. Extracellular Vesicles 4, 27497. 10.3402/jev.v4.27497 26320941 PMC4553261

[B24] Sundararaman B. Zhan L. Blue S. M. Stanton R. Elkins K. Olson S. (2016). Resources for the Comprehensive Discovery of Functional RNA Elements. Mol. Cel 61, 903–913. 10.1016/j.molcel.2016.02.012 PMC4839293 26990993

[B25] Tosar J. P. Cayota A. Eitan E. Halushka M. K. Witwer K. W. (2017). Ribonucleic Artefacts: Are Some Extracellular RNA Discoveries Driven by Cell Culture Medium Components? J. Extracell. Vesicles 6, 1272832. 10.1080/20013078.2016.1272832 28326168 PMC5328325

[B26] Tosar J. P. Rovira C. Cayota A. (2018a). Non-coding RNA Fragments Account for the Majority of Annotated piRNAs Expressed in Somatic Non-gonadal Tissues. Commun. Biol. 1, 2. 10.1038/s42003-017-0001-7 30271890 PMC6052916

[B27] Tosar J. P. Gámbaro F. Darré L. Pantano S. Westhof E. Cayota A. (2018b). Dimerization Confers Increased Stability to Nucleases in 5′ Halves from glycine and Glutamic Acid tRNAs. Nucleic Acids Res. 46, 9081–9093. 10.1093/nar/gky495 29893896 PMC6158491

[B28] Tosar J. P. García-Silva M. R. Cayota A. (2021). Circulating SNORD57 rather Than piR-54265 Is a Promising Biomarker for Colorectal Cancer: Common Pitfalls in the Study of Somatic piRNAs in Cancer. RNA 27, 403–410. 10.1261/rna.078444.120 33376191 PMC7962485

[B29] Van Nostrand E. L. Freese P. Pratt G. A. Wang X. Wei X. Xiao R. (2020). A Large-Scale Binding and Functional Map of Human RNA-Binding Proteins. Nature 583, 711–719. 10.1038/s41586-020-2077-3 32728246 PMC7410833

[B30] von Felden J. Garcia-Lezana T. Dogra N. Gonzalez-Kozlova E. Ahsen M. E. Craig A. (2021). Unannotated Small RNA Clusters Associated with Circulating Extracellular Vesicles Detect Early Stage Liver Cancer. Gut. 10.1136/gutjnl-2021-325036 PMC8795201 34321221

[B31] White B. S. Gentles A. J. de Reyniès A. Newman A. M. Lamb A. Heiser L. (2019). “A Tumor Deconvolution DREAM Challenge: Inferring Immune Infiltration from Bulk Gene Expression Data [abstract],” in Proceedings of the American Association for Cancer Research Annual Meeting 2019, Atlanta, GA. Philadelphia (PA), Mar 29-Apr 3, 2019.

